# The effect of repeated sprints on immunological modulation and the role of fat-free mass, biological maturation and dietary inflammatory index in male athletes: a quasi-experimental study with insights for training loads control

**DOI:** 10.3389/fspor.2025.1662761

**Published:** 2025-09-23

**Authors:** Paulo F. de Almeida-Neto, E. Alana D. Fernandes, Gilmara G. de Assis, Katsuhiko Suzuki, Roberto F. da Costa, Lívia de Melo Atanásio, Vanessa Carla Monteiro Pinto, Felipe J. Aidar, Breno Guilherme de Araújo Tinôco Cabral, Paulo Moreira Silva Dantas

**Affiliations:** ^1^ Health Sciences Center, Federal University of Rio Grande do Norte. Natal, Rio Grande do Norte, Brazil; ^2^ Escola Superior Desporto e Lazer, Instituto Politécnico de Viana do Castelo, Rua Escola Industrial e Comercial de Nun’Álvares, Viana do Castelo, Portugal; ^3^ Sport Physical Activity and Health Research & Innovation Center, Viana do Castelo, Portugal; ^4^ Faculty of Sport Sciences, Waseda University, Tokyo, Japan; ^5^ Faculty of Health Sciences, Universidad Autónoma de Chile, Providencia, Chile; ^6^ Federal University of Sergipe, UFS, São Cristovão, Brazil

**Keywords:** cytokines, inflammation, high-intensity exercise, sport, diet

## Abstract

**Background:**

Repeated sprint exercise (RSE) induces inflammation, which may be modulated by fat-free mass (FFM), biological maturation (BM), and dietary patterns, assessed by the Dietary Inflammatory Index (DII).

**Aim:**

To examine the influences of FFM, BM, and DII on cytokine responses to RSE in male athletes.

**Methods:**

A study with a quasi-experimental approach and cross-sectional design with a sample of 30 male athletes (20-adolescents, 10-adults). Blood samples were collected pre-, immediately after, 2 h and 24 h after RSE (3-sets of 6 × 35-m sprints). IL-1β, IL-6, IL-8, and IL-10 were analyzed via flow cytometry. FFM was assessed by DXA, BM by predictive models, and DII by 24 h dietary recalls. Prior exploratory analyses included Spearman's and partial correlations, and Mann–Whitney-*U*-tests. Main analyses were conducted using Generalized-Linear-Mixed-Models (GLMM).

**Results:**

The GLMMs confirmed that BM, FFM, and DII significantly influenced cytokine responses (*p* < 0.05). FFM emerged as a significant predictor of IL-1β (*p* = 0.0023). For IL-6, there was a time effect (*p* < 0.001) and a Time × BM interaction (*p* = 0.040), with FFM and DII being significant predictors in both groups. A similar interaction was observed for IL-8 (*p* = 0.036). For IL-10, there was a Time × BM interaction (*p* < 0.001), where adults showed superiority over adolescents (*p* < 0.05). *Post hoc* analyses revealed that adolescents with lower FFM had a more prolonged inflammatory response (increased-IL-6), while adults with higher FFM demonstrated a more effective anti-inflammatory capacity (increased-IL-10).

**Conclusion:**

FFM, BM, and DII play key roles in shaping the inflammatory response to RSE and should be considered when prescribing training loads to optimize recovery and performance.

## Graphical Abstract

**Figure:**
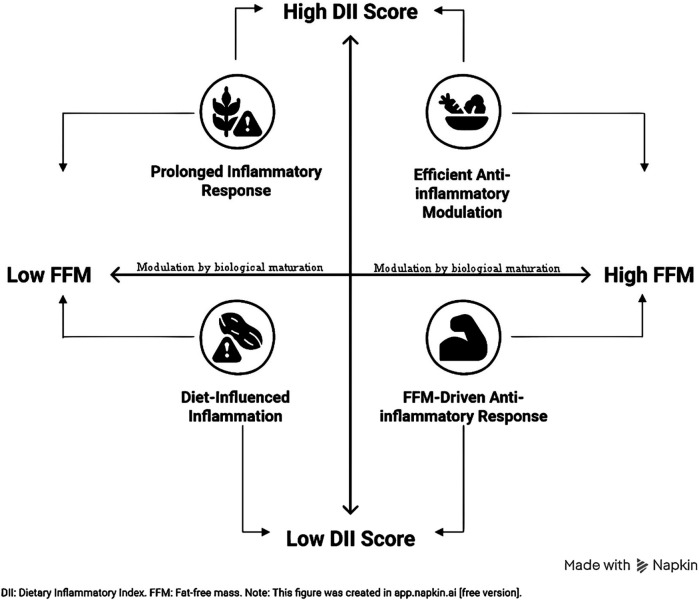

## Highlights

• Fat-free mass (FFM) and Dietary Inflammatory Index (DII) are significant predictors of post-exercise cytokine responses. FFM influences both pro-inflammatory (IL-6 & IL-8) and anti-inflammatory (IL-10) responses, while DII is associated with a more efficient modulation of the immune response.
• Biological maturation is a key determinant of the immune response to exercise. Less mature athletes (adolescents with lower FFM) exhibit a more prolonged inflammatory response, whereas more mature athletes (adults with higher FFM) demonstrate a more effective and acute anti-inflammatory capacity.

## Introduction

Fat-free mass (FFM), particularly, the skeletal muscle tissue plays an essential role in immunoinflammatory regulation through the expression of myokines. In fact, the muscle is considered an endocrine organ due to an increased release of such myokines as interleukin-6 (IL-6) and interleukin-10 (IL-10) (1), also known as exerkines, in response to mechanical and metabolic stress induced by high-intensity exercises (2, 3). IL-6 is involved in metabolic adaptations and contribute to inflammatory processes, while IL-10 essentially exerts an anti-inflammatory function that supports muscle recover (4, 5).

Athletes involved in intermittent sports, such as soccer, basketball, and handball, are frequently exposed to repeated high-intensity efforts interspersed with brief recovery periods (6, 7). This type of physical demand elicits substantial metabolic and mechanical stress, which in turn activates immune and inflammatory pathways, including the release of myokines (8, 9). Previously, it was evidenced that the sport discipline (jiu-jitsu, soccer, and volleyball) did not exert a significant influence on the immune responses derived from repeated sprint exercise (RSE) in adolescent athletes of intermittent sports, suggesting that the physiological demands at the immunological level are similar across different intermittent sports (10).

These athletes are particularly susceptible to acute and chronic inflammatory responses due to the cyclical nature of training and competition, which may influence performance, recovery, and injury risk (9). Understanding the inflammatory milieu in this context is critical, especially because the regulation of pro- and anti-inflammatory cytokines can impact the balance between adaptation and overtraining (11).

The RSE is a high intensity short training strategy for athletes engaged in intermittent sports (12, 13). We have previously shown that RSE induces an increase in leukocyte counts and the neutrophil-to-lymphocyte ratio in adolescents for up to two hours after the exercise, suggesting its modulatory effect on inflammatory processes (10). Also, such effect seems to be even higher in adults (8). This led to the sense that the biological maturation (BM) might be a modulator of immune responses to RSE (10, 14). However, inflammatory responses may be influenced by several other factors like dietary patterns (15). The BM status is determined by various markers associated with physiological mechanisms that promote increases in the total FFM, especially during adolescence (16), like secondary sexual characteristics (pubertal stages), peak height velocity (PHV), and the degree of epiphyseal ossification (skeletal maturation) (14). Thus, it is possible that adolescent muscle production of myokines is influenced by BM stages.

However, to investigate how FFM levels can modulate exercise-induced myokine release it is necessary to control both BM and dietary patterns. This can be achieved through subgroup analyses based on the levels of FFM and dietary patterns or through appropriate statistical techniques such as partial Pearson correlation or analysis of covariance (17, 18). FFM is determined using dual-energy x-ray absorptiometry (DXA) (19), and dietary patterns can be evaluated through a 24-h dietary recalls (24h-R) of the Dietary Inflammatory Index (DII) (20). It is also possible to verify relationships among these variables in adults and observe the behavior of patterns in adolescents undergoing BM to examine how DII may modulate the effect of FFM on myokine release after RSE in adults.

Although a previous study (21) by our group investigated the immune response to RSE in athletes of different age groups, it did not address the influence of critical covariates such as FFM and the DII on cytokine modulation. The present study advances the knowledge in the field by uniquely analyzing the role of these variables, particularly DII (22), an index of easy clinical use for sports nutrition, which can offer valuable insights into the modulation of inflammatory responses in athletes of intermittent sports.

Considering that the inflammatory response to exercise is relevant for adjusting training volume, intensity, and frequency (14), and that the understanding of factors that modulate mytokine release—such as FFM, BM, and DII— offers valuable insights for training management in sports; we hypothesized that the relationship between FFM and RSE-induced myokine release is modulated by BM and dietary patterns in adolescents. Therefore, the aim of this study was to analyze the influence of FFM, BM, and DII on myokine release after RSE in male athletes, and to identify inflammatory markers that can guide decision-making in training loads control.

## Methods

A study with a quasi-experimental approach and cross-sectional design, was conducted with a sample of 30 male athletes, including 20 adolescents [Age: 12.9 ± 1.1 years; PHV = −0.7 ± 1.1; Puberty score = 0.7 ± 1.4; Skeletal maturation = 0.8 ± 1.2; Total body mass (kg) = 45.5 ± 16.7; FFM (kg) = 34.7 ± 10.2] and 10 adults [Age: 23.2 ± 2.1 years; Total body mass (kg) = 69.9 ± 9.9; FFM (kg) = 56.1 ± 6.0]. All participants were athletes involved in intermittent sports (soccer, volleyball, basketball, karate, and jiu-jitsu) and regularly participated in regional and national competitions (Tier 3/4) (23). We emphasize that intermittent sports have similar physiological demands, which is why the sample can be treated as a homogeneous group from this perspective. In addition, all participants reported a weekly training frequency of 4–6 days, with daily sessions lasting between 2 and 4 h. They were also duly affiliated with the federations of their respective sports and ranked among the top 20 athletes at the regional level in their categories.

The participants were voluntarily recruited from sports clubs in the city of Natal (Northeast Brazil, state of *Rio Grande do Norte*). To be included in the sample, individuals were required to meet the following criteria: (a) engage in at least two training sessions per week, each lasting a minimum of 60 min, for at least six months prior to the study; (b) participate in intermittent sports; and (c) have a competition history at the regional, national, or international level for at least one year. Exclusion criteria included: presence of upper respiratory tract infection symptoms, use of supplements with potential effects on the immune system (such as vitamin C, glutamine, among others), diagnosis of clinical diseases, or musculoskeletal injuries within the six months preceding the study.

The study was approved by the Ethics Committee of the Federal University of Rio Grande do Norte (#35197020.8.0000.5537) and complies with the Declaration of Helsinki. The study was registered in the Open Science Framework Registries (https://doi.org/10.17605/OSF.IO/NPZQX). All participants, and their legal guardians in the case of minors, were informed about the procedures and signed the consent or assent form, as applicable.

### Procedures

**Figure 1:**
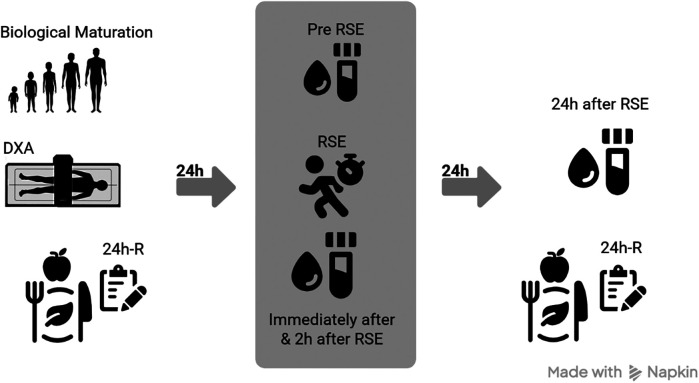
Procedures. DXA, dual-energy x-ray absorptiometry; 24h-R, 24-h dietary recall; RSE, repeated sprint exercise. This figure was created in https://www.app.napkin.ai [free version].

On the first day of the protocol, participants underwent anthropometric and body composition assessments to estimate BM and quantify FFM levels. At this time, the first 24h-R was also administered to characterize the participants' dietary pattern prior to the intervention. After a 24-h interval, resting blood samples were collected. Subsequently, participants were taken to an official athletics track, where they performed a brief five-minute warm-up consisting of jumps and short walks. This was followed by the RSE protocol, which consisted of three sets of six sprints, with 10 s of passive rest between sprints and five minutes of passive rest between sets. Additional blood samples were collected immediately after the RSE, as well as 2 h and 24 h after exercise, to analyze serum cytokine levels at different time points (pre, immediately after, 2 h, and 24 h after RSE). It is worth noting that, at the 24-h after protocol time point, participants completed the second 24h-R, allowing for the monitoring of dietary intake during the after-intervention period as well (Figure 1).

To ensure blinding regarding the implementation of the RSE protocol, the intervention was conducted by an external collaborator not affiliated with the research team, thereby guaranteeing impartiality at this stage of the study. The 24h-R were administered by a registered dietitian who was also external to the research team. Blood sample collection was carried out by an independent professional, ensuring the neutrality of the procedure. During the laboratory analyses of cytokines, all data were coded to conceal participant identities, preventing evaluators from knowing the origin of each sample. Similarly, during statistical analyses, the data remained masked, making it impossible to identify the groups or individual participants. These measures reinforced the objectivity and integrity of the results obtained.

### Anthropometry

Body mass was measured using a Filizola® digital scale with a 200 kg capacity and 0.10 kg precision (São Paulo, Brazil). For this assessment, participants were barefoot and wore light clothing. Height was measured using a Sanny® stadiometer with a precision of 0.1 mm (São Paulo, Brazil). All measurements were taken by a single evaluator following the protocols of the International Society for the Advancement of Kinanthropometry (ISAK) (24). The intra-observer technical error for anthropometric measurements was ≤1.0% (25). FFM levels were assessed using dual-energy x-ray absorptiometry (DXA) with a LUNAR®/GE PRODIGY - LNR 41.990 device (Washington, DC, USA), employing the enCORE software, GE Healthcare®, version 15.0 (Madison, WI, USA). For adolescent participants, population-specific algorithms were applied (26). During the assessments, the DXA device operated under the following configuration: whole-body scan; voltage (kV): 76.0; current (mA): 0.150; radiation dose (µGγ): 0.4 (very low, with no health risk).

### Biological maturation profile

The BM profile was assessed through estimates of peak height velocity (PHV), puberty scores, and skeletal maturity. PHV stage was determined using the mathematical model proposed by Moore et al. (27), for males aged between 8 and 18 years: PHV = *−8.128741* *+* *[0.0070346* *×* *Age[years]* *×* *Sitting height(cm)]*, where: (cm) = centimeters. Based on the outcome of this model, PHV classification was defined as: Pre PHV (<−1), Circum PHV (entre −1 e 1) e Post PHV (>1). The puberty score was calculated using the model proposed by Almeida-Neto et al. (28), for males aged between 6 and 18 years: *Puberty (score)* *=* *−17.357* *+* *(0.603* *×* *Age(years)]* *+* *[0.127* *×* *Sitting height(cm)]*. From these scores, Tanner stages were classified as follows: I ≤ −1.815; II = −1.816 to −0.605; III = −0.606–0.695; IV = 0.696–3.410 & V > 3.410. Stage I corresponds to the prepubertal phase, stages II to IV correspond to the pubertal phase, and stage V represents the postpubertal phase (28). Skeletal age was determined based on the mathematical model proposed by Cabral et al. (29): *Skeletal age* *=* *−11.620* *+* *7.004* *×* *(Height_(m)_)* *+* *1.226* *×* *(Dsex)* *+* *0.749* *×* *(Age_(years)_) - 0.068* *×* *(Triceps skinfold_(mm)_)* *+* *0.214* *×* *(Corrected arm circumference_(cm)_) - 0.588* *×* *(Humerus diameter_(cm)_)* *+* *0.388* *×* *(Femoral diameter_(cm)_)*, where, Dsex: For male sex = 0; for female sex = 1. (m): meters. (mm): millimeters. (cm): centimeters. The corrected arm circumference was calculated using the formula: *Corrected Arm Circumference_(cm)_* *=* *Contracted biceps circumference_(cm)_ - (Triceps skinfold_(mm)_/10)*, where, (mm): millimeters. (cm) centimeters. After determining skeletal age, skeletal maturity was classified using the equation proposed by (30): *Skeletal maturity* *=* *Skeletal Age – Chronological age*. Based on this difference, skeletal maturation was categorized as follows: Delayed (<−1.0), Synchronous (between −1.0 and 1.0), and Accelerated (>1.0).

### Upper respiratory tract infection symptoms

On the day of the RSE protocol, prior to the first blood collection, upper respiratory tract infection symptoms (URTIS) were assessed using the Wisconsin Upper Respiratory Symptom Survey-21 (WURSS-21) questionnaire (31, 32). Only volunteers who showed no signs of URTIS were retained as participants in the study. It is worth noting that this tool has been previously used in a study involving young athletes and validated for pediatric populations (33, 34). In the present study, none of the participants needed to be excluded due to URTIS symptoms on the day of RSE.

### Physical activity level

The physical activity level was assessed using the web-based version of the International Physical Activity Questionnaire (IPAQ) (33), validated for estimating metabolic equivalents (METs) related to energy expenditure from physical activities (35). The questionnaire covers activities at work, during transportation, household tasks, and leisure/sports. The web-IPAQ automatically calculates METs for habitual energy expenditure, providing scores for each domain and a total score. Based on total METs, the web-IPAQ classifies physical activity level as low, moderate, or high. Participants completed the web-IPAQ in the presence of at least one researcher and, in the case of adolescents, with a guardian to assist with interpreting the questions. All participants in the present study reported a high level of physical activity.

### Sleep pattern

Sleep patterns were assessed using a sleep diary, in which participants recorded the time they went to bed, perceived time to fall asleep, wake-up time, total time in bed, and whether there were interruptions during the night (36). Based on these entries, sleep efficiency was calculated (considered good when >85%) (36). Records were made on the day of the RSE (referring to the previous night) and on the day following the RSE (referring to the intervention night). All participants in this study presented sleep efficiency between 85% and 92%.

### Dietary pattern and dietary inflammatory Index (DII)

The dietary pattern was assessed using two 24h-R, applied on non-consecutive days, including one typical day and one atypical day (37). To estimate habitual intake, the *Brazilian Food Composition Table* (38) and the virtualized *Multiple Source Method* (version 1.0.1.87) were used to adjust for intra- and interpersonal variability (39). The inflammatory potential of the diet was measured using the DII, which quantifies the pro- or anti-inflammatory effect of diet based on scores associated with inflammatory biomarkers and health outcomes (40).

The DII calculation was based on the regional dietary pattern of the city of Natal (Northeast Brazil), where the participants of the present study live and train. The specific version used was characterized and validated by the BRAZUCA study (22), which includes 35 of the 45 items from the original DII model described by Shivappa et al. (41). It is noteworthy that the 10 additional items from the original model were not assessed in the 24h-R analyses of the participants in this study, aligning with the specific parameters adapted from the BRAZUCA study (22).

Each parameter was assigned a score of +1 (pro-inflammatory), −1 (anti-inflammatory), or 0 (neutral). The DII score was calculated based on the Z-score of individual intake, converted to a centered percentile and weighted by the nutrient's inflammatory effect score. The global DII corresponds to the sum of the individual parameter values and is interpreted as follows: anti-inflammatory diet (<0) or pro-inflammatory diet (≥0) (42). Details of the DII analysis are provided in Supplementary Material 1 (Data spreadsheet).

### Repeated sprint exercise

In the 24 h prior to the RSE protocol, participants were instructed to refrain from engaging in high-intensity physical activities. The RSE involved 18 high-intensity sprints performed on an official athletics track, under an average temperature of 26.8 ± 0.8°C. The protocol was divided into three sets, each consisting of six 35-m sprints with 10 s of passive rest between repetitions. Between sets, participants were given a 5-min passive rest period, during which they remained seated on chairs with back support and knees flexed at 90°. During the RSE, participants received verbal encouragement, and the rest intervals between each sprint were monitored by two timekeepers. A third timekeeper monitored the rest periods between sets. All timekeepers used digital stopwatches (TecTime®, São Paulo, Brazil). Participants were instructed to sprint at maximal effort to ensure the high intensity of the exercise.

The Rating of Perceived Exertion (RPE) scale, proposed by Borg (43), was used to assess the subjective intensity of the RSE. The scale was applied immediately after the final sprint. This visual, monochromatic scale ranges from 6 to 20, where 6 corresponds to complete rest and 20 to maximal effort. Participants underwent prior familiarization with the RPE scale 72 h before the RSE and were re-familiarized on the day of the protocol. All participants reported RPE scores between 17 and 20, indicating a high-intensity effort during RSE.

### Blood analyses

At each time point in the present study (pre, immediately after, 2 h, and 24 h after RSE), 10 ml of peripheral blood was collected from the antecubital vein using the vacuum method. The blood was stored in dry tubes (BD-Vacutainer, 5.4 mg Plus Plastic) and processed for laboratory analysis. We analyzed the serum concentrations of the cytokines [interleukin (IL)] IL-1β, IL-6, IL-8 and IL-10 (44). For this purpose, peripheral blood samples were assessed by flow cytometry using a BD Biosciences® device (Model: BD FACSCanto™ II Flow Cytometer, Serial Number: V96100619, New York, USA), with the BD™ Cytometric Bead Array (CBA) Human Inflammatory Cytokines kit (São Paulo, Brazil). All procedures followed the manufacturer's instructions provided in the kit manual (45). After flow cytometry, the data were processed using BD Biosciences® FCAP Array™ software (Version 3.0; New York, USA).

### Statistical analysis

All statistical analyses were performed using the open-source software JAMOVI® (version 2.3, Sidney, Australia), and figures were produced using GraphPad Prism® (version 8.01, California, USA). The significance level was set at *p* < 0.05.

#### Preliminary non-parametric analyses

Data distribution was assessed using the Shapiro–Wilk test, skewness and kurtosis coefficients (acceptable range: −1.96 to +1.96), and visual inspection of Q-Q plots. Since assumptions of normality were violated, initial analyses relied on non-parametric tests as an exploratory step.

Associations between FFM and cytokine concentrations were evaluated using Spearman's rank correlation coefficient (*ρ*). To account for potential confounders, partial correlations were conducted, controlling for BM in adolescents (puberty score, PHV, and skeletal maturity) and for the DII in both adolescents and adults. Ninety-five percent confidence intervals (95% CI) for Spearman's coefficients were estimated through bias-corrected bootstrapping with 1,000 resamples (46). The magnitude of Spearman's *ρ* was interpreted as (47): insignificant (<0.10), weak (0.10–0.39), moderate (0.40–0.69), strong (0.70–0.89), and very strong (0.90–1.00).

For group comparisons, participants were categorized by median split into lower vs. higher subgroups for FFM, DII, and BM stage, separately within adolescents and adults. Percent changes in cytokine concentrations (Δ%) across time points were compared using the Mann–Whitney *U*-test. Effect sizes were reported as rank-biserial correlation coefficients (48) and interpreted as (49): small (≤0.05), medium (0.06–0.25), large (0.26–0.50), or very large (>0.50).

Samples were categorized based on the median split of the following variables of interest:

• FFM (lower vs. higher);
• DII (lower vs. higher);
• Stage of BM [Pre-PHV (lower mature) vs. Circum/Post-PHV (higher mature)].

We emphasize that age groups were considered in this process, as described below: (i) adolescents with lower levels vs. adolescents with higher levels; (ii) adults with lower levels vs. adults with higher levels. Thus, comparisons were made within each age group.

Thus, when grouping the subjects for the sub-analyses, the division was balanced: 50% for *lower levels* and 50% for *higher levels*, as shown below:

• Adolescents FFM lower (*n* = 10) vs. Adolescents FFM higher (*n* = 10);
• Adults FFM lower (*n* = 05) vs. Adults FFM higher (*n* = 05);
• Adolescents DII lower (*n* = 10) vs. Adolescents DII higher (*n* = 10);
• Adults DII lower (*n* = 05) vs. Adults DII higher (*n* = 05);
• Adolescents BM lower (*n* = 10) vs. Adolescents BM higher (*n* = 10).

Subsequently, percent changes in cytokine levels (Δ%) between subgroups were compared. Δ% was calculated as:

Δ%=[(AfterRSEvalue/PreRSEvalue)–1]×100

#### *Post hoc* statistical power

Since 16 correlation analyses were performed between FFM and various cytokines across four time points (pre, immediately after, 2 h, and 24 h after RSE), Bonferroni correction was applied to control for type I error. The adjusted alpha level (adjusted p) was set at *<0.003125* for correlations.

Similarly, 12 subgroup comparisons were conducted for Δ% values across three after RSE time points (immediately after, 2 h, and 24 h). For these comparisons, the adjusted *p*-value was defined as *<0.0042*.

*Post hoc* power analyses were conducted for the main significant findings (based on the adjusted *p*-values), indicating statistical power greater than 0.80 for effects with r ≥ 0.66. This provides greater confidence in the inferences drawn in these cases. Power analyses were performed using G*Power software (version 3.1, Düsseldorf, Germany). Nevertheless, caution is advised when interpreting subgroup results with small samples, such as the adult group, due to lower statistical power.

#### Main analysis: generalized linear mixed models (GLMM)

Given the longitudinal and repeated-measures nature of the dataset (four time points per participant: pre, immediately after, 2 h, and 24 h after RSE), the small and unbalanced sample size (20 adolescents, 10 adults), and the need to control for covariates, the primary analyses were performed using Generalized Linear Mixed Models (GLMM). This approach allowed us to: (i) leverage the within-subject design, (ii) adjust for individual differences in FFM and DII, and (iii) provide greater robustness against violations of normality and sample imbalance.

GLMMs were implemented in JAMOVI® using the GAMLj module. For each cytokine (IL-1β, IL-6, IL-8, IL-10), log-transformed concentrations were specified as the dependent variable, assuming a Gamma distribution with log link function due to the skewness of cytokine data. The fixed-effects structure included:

• Time (four levels: pre, immediately after, 2 h and 24 h after RSE),
• Age group [BM] (adolescents vs. adults),
• FFM (standardized *z*-scores),
• DII (standardized *z*-scores),
• and the interaction Time × Age group.

Random effects were modeled at the participant level (ID), with random intercepts and slopes for time (1 + Time | ID), thereby accounting for intra-individual variability. Estimation was based on restricted maximum likelihood (REML), and convergence was checked across multiple optimizers (*bobyqa*, *Nelder-Mead*, *nloptwrap*).

In our study, the repeated-measures design, with data collection at four time points, allowed us to use each athlete as their own control. The analysis using GLMM was specifically adopted to model intra-subject effects over time, mitigating the risks associated with the absence of a control group.

#### Model outputs and *post hoc* testing

For each model, type III Wald chi-square tests were used to evaluate omnibus fixed effects. Fixed-effect parameter estimates (B), standard errors (SE), *z*-values, *p*-values, and 95% confidence intervals were reported. Estimated marginal means (EMMs) were calculated, and pairwise comparisons between time points were adjusted using Bonferroni correction. Interaction effects (Time × Age group) were explored through simple-effects analyses, keeping other covariates constant at their means.

## Results

In the total sample analysis, a positive correlation was identified between FFM and IL-6 levels both pre and immediately after RSE, as well as a negative correlation with IL-10 at 24 h after RSE (Table 1). Among adolescents, a positive association was found between FFM and IL-6 at 2 h after RSE, while negative correlations were observed with IL-6 and IL-8 at 24 h after RSE.

**Table 1:** Correlation analysis of FFM levels with cytokine levels considering the interference of BM in adolescent athletes.

Cytokine	Total sample (*n* = 30)				Adolescent (*n* = 20)								Adult (*n* = 10)			
									Controlling BM markers (partial correlation)							
	*ρ*	*p*	CI 95%		*ρ*	*p*	CI 95%		*ρ*	*p*	CI 95%		*ρ*	*p*	CI 95%	
	FFM Kg		Bootstraps (1.000)		FFM Kg		Bootstraps (1.000)		FFM Kg		Bootstraps (1.000)		FFM Kg		Bootstraps (1.000)	
	Pre Repeated Sprints Exercise				Pre Repeated Sprints Exercise				Pre Repeated Sprints Exercise				Pre Repeated Sprints Exercise			
IL-1β (pg/ml)	0.148	0.444	−0.232	0.493	0.118	0.619	−0.365	0.547	−0.184	0.480	−0.735	0.473	0.209	0.562	−0.691	0.858
IL-6 (pg/ml)	0.423*	0.0022	0.059	0.699	0.143	0.548	−0.301	0.583	0.200	0.441	−0.593	0.806	0.042	0.919	−0.774	0.805
IL-8 (pg/ml)	0.358	0.057	−0.024	0.629	−0.301	0.197	−0.665	0.150	−0.116	0.657	−0.569	0.603	−0.164	0.657	−0.789	0.585
IL-10 (pg/ml)	−0.034	0.862	−0.401	0.298	0.292	0.212	−0.159	0.674	0.508*	0.003	−0.138	0.844	−0.752*	0.0012	−0.988	−0.229
	Immediately after Repeated Sprints Exercise				Immediately after Repeated Sprints Exercise				Immediately after Repeated Sprints Exercise				Immediately after Repeated Sprints Exercise			
IL-1β (pg/ml)	0.203	0.290	−0.153	0.534	0.199	0.400	−0.284	0.628	−0.254	0.325	−0.690	0.572	0.486	0.154	−0.261	0.946
IL-6 (pg/ml)	0.377*	0.003	0.039	0.639	0.132	0.580	−0.287	0.584	0.233	0.368	−0.606	0.798	−0.006	1.000	−0.689	0.699
IL-8 (pg/ml)	0.328	0.083	−0.037	0.615	−0.003	0.992	−0.479	0.522	0.142	0.587	−0.386	0.660	−0.079	0.838	−0.673	0.801
IL-10 (pg/ml)	0.209	0.276	−0.146	0.556	0.331	0.154	−0.047	0.628	0.196	0.450	−0.520	0.666	−0.673*	0.003	−0.925	−0.118
	2 h after Repeated Sprints Exercise				2 h after Repeated Sprints Exercise				2 h after Repeated Sprints Exercise				2 h after Repeated Sprints Exercise			
IL-1β (pg/ml)	0.065	0.738	−0.392	0.450	0.321	0.168	−0.192	0.777	−0.246	0.342	−0.661	0.462	0.328	0.354	−0.401	0.852
IL-6 (pg/ml)	0.356	0.058	−0.050	0.693	0.619*	0.003	0.202	0.920	0.710*	0.001	−0.014	0.922	0.745*	0.0018	0.150	1.000
IL-8 (pg/ml)	0.333	0.078	−0.068	0.668	0.014	0.955	−0.508	0.552	0.261	0.312	−0.367	0.730	0.333	0.349	−0.400	0.889
IL-10 (pg/ml)	0.009	0.964	−0.306	0.353	0.239	0.310	−0.255	0.702	0.371	0.143	−0.307	0.794	0.143	0.694	−0.754	0.800
	24 h after Repeated Sprints Exercise				24 h after Repeated Sprints Exercise				24 h after Repeated Sprints Exercise				24 h after Repeated Sprints Exercise			
IL-1β (pg/ml)	−0.091	0.639	−0.510	0.364	0.012	0.960	−0.459	0.514	−0.552*	0.0022	−0.843	0.216	0.334	0.345	−0.368	0.805
IL-6 (pg/ml)	−0.105	0.588	−0.486	0.267	−0.495*	0.0026	−0.803	−0.092	−0.160	0.540	−0.609	0.480	−0.061	0.868	−0.700	0.676
IL-8 (pg/ml)	−0.142	0.462	−0.521	0.198	−0.534*	0.0015	−0.845	−0.079	−0.214	0.409	−0.645	0.578	−0.333	0.349	−0.837	0.358
IL-10 (pg/ml)	−0.440*	0.0017	−0.739	−0.003	−0.136	0.568	−0.573	0.308	−0.135	0.607	−0.664	0.491	−0.681*	0.0030	−0.962	−0.038

ρ, Spearman Rho coeficient; FFM, fat-free mass; IL, interleukin; β, Beta; Rho, Spearman's correlation coefficient; CI, confidence interval; BM, biological maturation.

* Statistically significant (Bonferroni correction *p*-adjusted *<0.003125*).

When adjusting for BM profile markers (puberty score, PHV, and skeletal maturity) within the adolescent group, the following were observed: a positive correlation between FFM and IL-10 pre RSE; a positive correlation with IL-6 at 2 h after RSE; and a negative correlation with IL-1β at 24 h after RSE (Table 1).

Among adults, FFM showed a negative correlation with IL-10 at three time points (pre, 2 h, and 24 h after RSE), and a positive correlation with IL-6 at 2 h after the protocol (Table 1).

After controlling for the effect of the DII in the correlation analyses between FFM and cytokine levels in the total sample, significant correlations were observed between FFM and IL-6 at the pre RSE time point, and between FFM and IL-10 at 24 h after RSE.

In the adult group, significant correlations were identified between FFM and IL-10 both immediately and 24 h after RSE. Additionally, after controlling for DII, no significant correlations were found between FFM and cytokine levels in the adolescent group (Table 2).

**Table 2:** Correlation analysis of FFM levels with cytokine levels considering the interference of the inflammatory index of the diet.

Cytokine	Total Sample (*n* = 30)				Adolescent (*n* = 20)				Adult (*n* = 10)			
	Controlling DII (Partial Correlation)				Controlling DII (Partial Correlation)				Controlling DII (Partial Correlation)			
	*ρ*	*p*	CI 95%		*ρ*	*p*	CI 95%		*ρ*	*p*	CI 95%	
	FFM Kg		Bootstraps (1.000)		FFM Kg		Bootstraps (1.000)		FFM Kg		Bootstraps (1.000)	
	Pre Repeated Sprints Exercise				Pre Repeated Sprints Exercise				Pre Repeated Sprints Exercise			
IL-1β (pg/ml)	0.155	0.4	−0.241	0.496	0.186	0.4	−0.218	0.520	0.522	0.1	−0.737	0.931
IL-6 (pg/ml)	0.441*	0.001	0.077	0.680	0.033	0.8	−0.258	0.418	−0.399	0.2	−0.851	0.776
IL-8 (pg/ml)	0.343	0.07	−0.091	0.608	−0.265	0.2	−0.666	0.045	−0.397	0.2	−0.895	0.804
IL-10 (pg/ml)	−0.015	0.9	−0.364	0.320	0.228	0.3	−0.148	0.574	−0.661	0.053	−0.981	−0.171
	Immediately after Repeated Sprints Exercise				Immediately after Repeated Sprints Exercise				Immediately after Repeated Sprints Exercise			
IL-1β (pg/ml)	0.178	0.3	−0.193	0.491	0.192	0.4	−0.123	0.524	0.588	0.09	−0.380	0.935
IL-6 (pg/ml)	0.361	0.054	0.045	0.630	0.109	0.6	−0.205	0.486	−0.321	0.4	−0.805	0.669
IL-8 (pg/ml)	0.327	0.08	−0.083	0.628	0.223	0.3	−0.387	0.651	−0.329	0.3	−0.805	0.932
IL-10 (pg/ml)	0.183	0.3	−0.245	0.582	0.295	0.2	−0.045	0.606	−0.677*	0.003	−0.919	−0.403
	2 h after Repeated Sprints Exercise				2 h after Repeated Sprints Exercise				2 h after Repeated Sprints Exercise			
IL-1β (pg/ml)	0.048	0.8	−0.319	0.430	−0.139	0.5	−0.403	0.330	0.623	0.07	−0.419	0.945
IL-6 (pg/ml)	0.347	0.06	−0.051	0.700	0.426	0.06	0.182	0.824	0.663	0.052	0.442	0.973
IL-8 (pg/ml)	0.338	0.07	−0.073	0.650	0.394	0.09	−0.307	0.910	0.324	0.4	−0.540	0.935
IL-10 (pg/ml)	0.02	0.9	−0.334	0.486	0.186	0.447	−0.268	0.604	0.274	0.5	−0.722	0.922
	24 h after Repeated Sprints Exercise				24 h after Repeated Sprints Exercise				24 h after Repeated Sprints Exercise			
IL-1β (pg/ml)	−0.087	0.6	−0.533	0.327	−0.342	0.1	−0.622	−0.126	0.660	0.051	−0.478	0.943
IL-6 (pg/ml)	−0.124	0.5	−0.472	0.256	−0.351	0.1	−0.627	0.013	−0.365	0.3	−0.877	0.640
IL-8 (pg/ml)	−0.136	0.5	−0.525	0.240	−0.435	0.06	−0.667	−0.195	−0.456	0.2	−0.888	0.475
IL-10 (pg/ml)	0.416*	0.003	−0.719	0.555	−0.119	0.6	−0.457	0.177	−0.700*	0.003	−0.951	−0.241

ρ, Spearman rho coeficient; DII, dietary inflammatory index; FFM, fat-free mass; IL, interleukin; β, beta; Rho, Spearman's correlation coefficient; CI, confidence interval; BM, biological maturation.

* Statistically significant (Bonferroni correction *p*-adjusted <0.003125).

**Figure 2:**
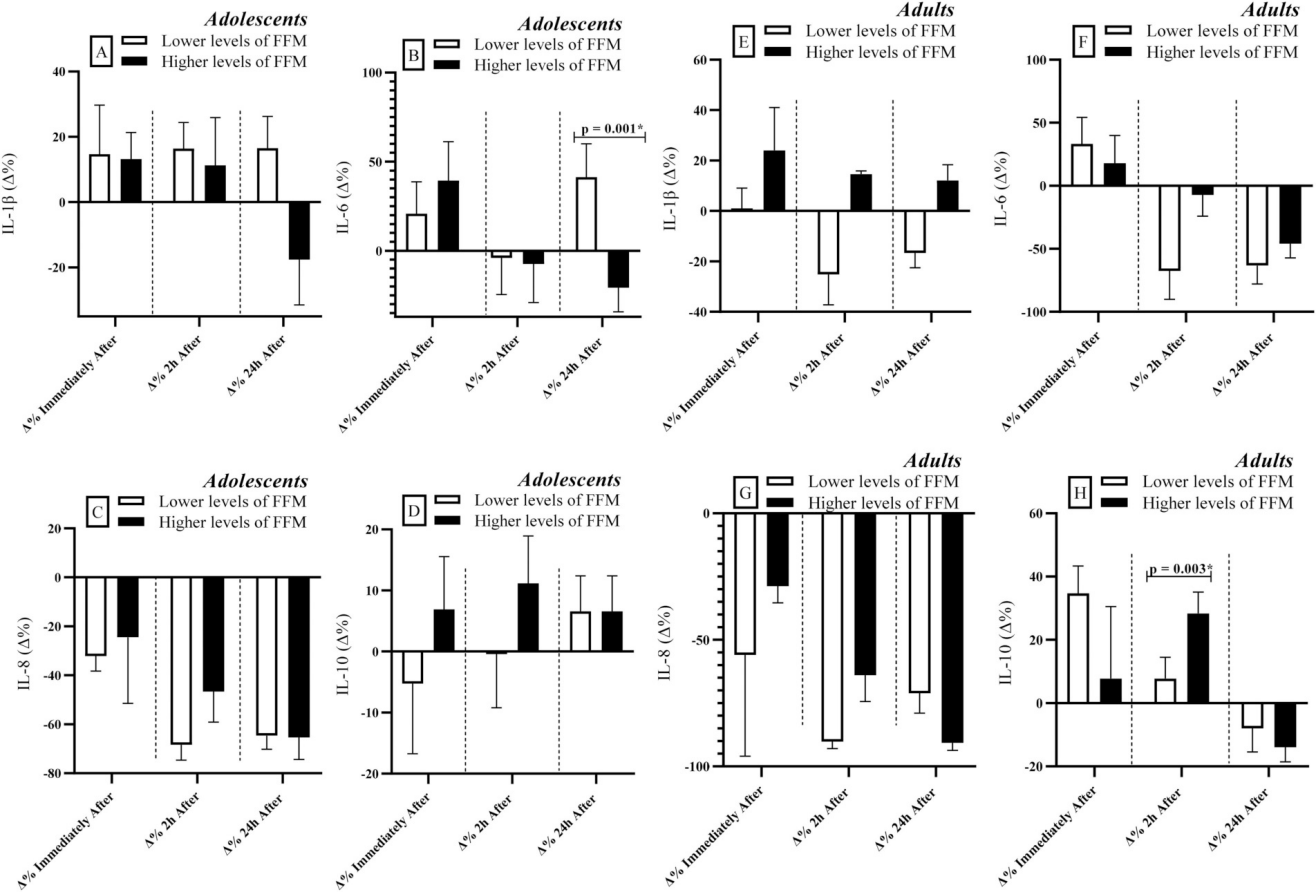
Comparisons of cytokine levels considering the division of groups based on FFM levels (lower vs. higher levels). FFM: Fat-free mass. IL: Interleukin. Δ: Delta Coefficient. *Statistically significant (Bonferroni correction *p*-adjusted <0.00417).

Adolescents with lower levels of FFM exhibited greater percent variation (Δ%) in IL-6 levels at 24 h after RSE [r = 0.66; Power = 0.81 (Figure 2B)], compared to their peers with higher FFM levels. No significant differences were observed between groups for the other cytokines (*p* > 0.05) (IL-1β, IL-8 & IL-10).

Among adults, individuals with higher FFM levels showed greater percent variation (Δ%) in IL-10 levels at 2 h after RSE [*r* = 0.84; Power = 0.88 (Figure 2H)] compared to those with lower FFM levels. As observed in the adolescent group, no significant differences were found between groups for the remaining cytokines analyzed (*p* > 0.05) (IL-1β, IL-6 & IL-8).

**Figure 3:**
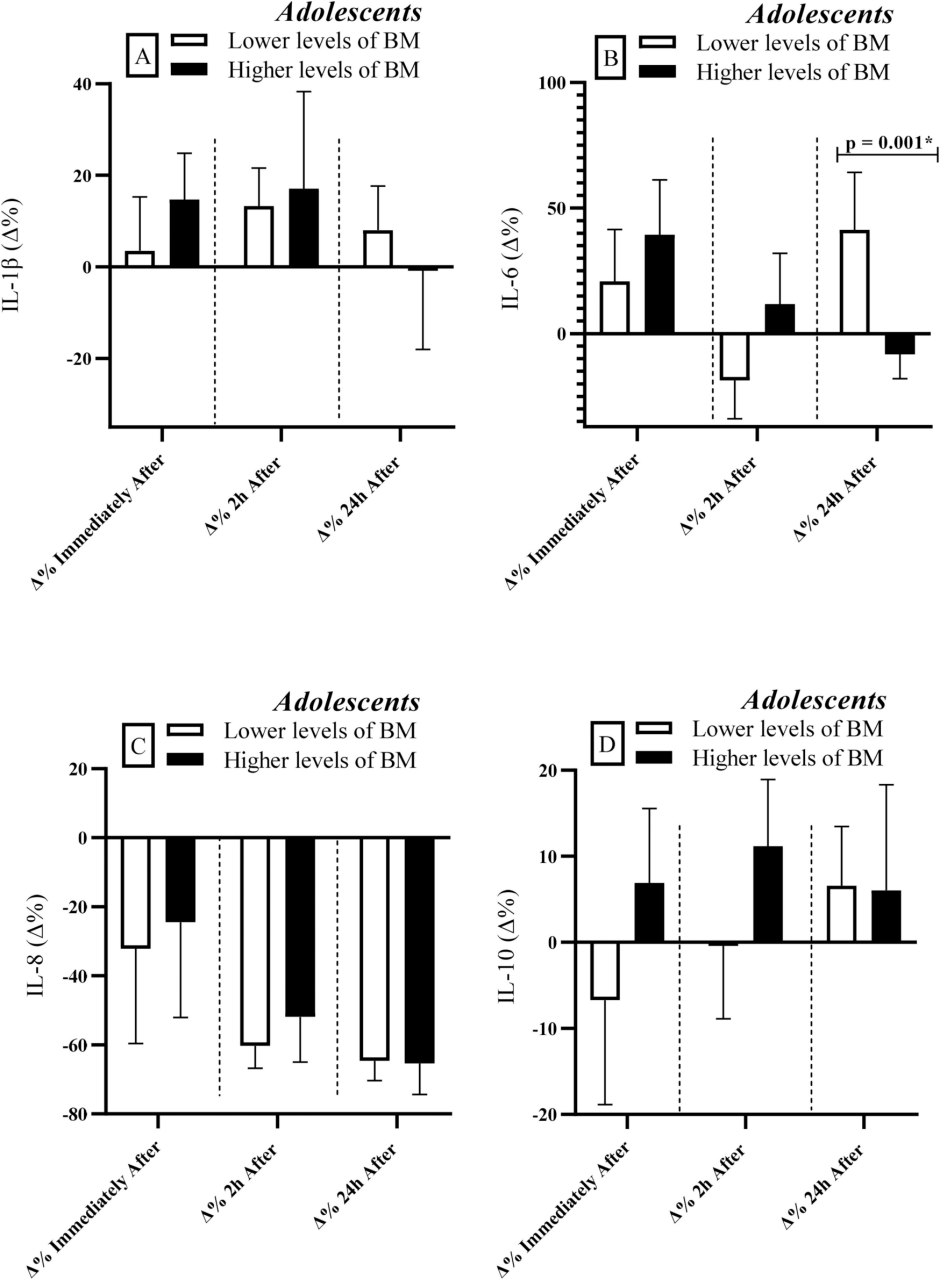
Comparisons of cytokine levels considering the division of the adolescent group based on BM levels (lower vs. higher levels). IL, interleukin; BM, biological maturation; Δ, delta coefficient. *Statistically significant (Bonferroni correction *p*-adjusted <0.00417).

Lower biologically mature adolescents exhibited greater percent variation (Δ%) in IL-6 levels at 24 h after RSE [*r* = 0.66; Power = 0.81 (Figure 3B)] compared to their more mature peers. No significant differences between groups were observed for the other cytokines (IL-1β, IL-8 & IL-10).

**Figure 4:**
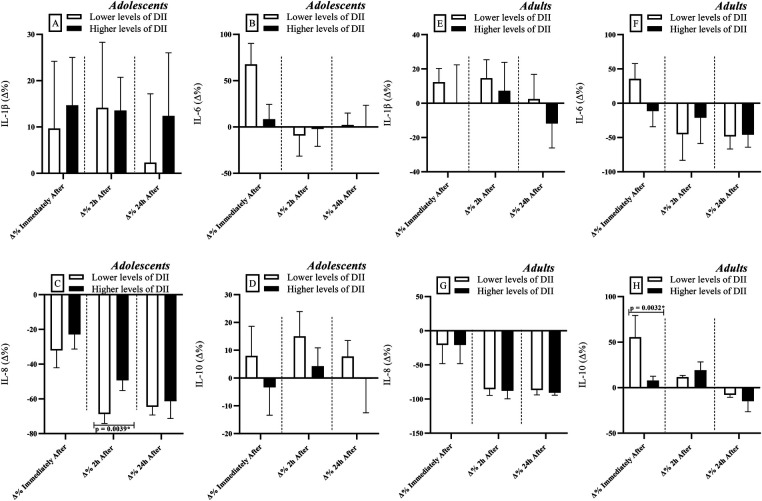
Comparisons of cytokine levels considering the division of groups based on dietary inflammatory index [DII] levels (lower vs. higher levels). IL, interleukin; Δ, delta coefficient. *Statistically significant (Bonferroni correction *p*-adjusted <0.00417).

When adolescents and adults were grouped according to DII levels, differences were observed only among adolescents at 2 h after RSE. At this time point, individuals with higher DII levels presented higher IL-6 concentrations compared to those with lower DII levels [*r* = 0.540; Power = 0.80 (Figure 4C)]. Additionally, in the adult group, individuals with lower DII levels exhibited higher IL-10 concentrations than their counterparts with higher DII levels immediately after RSE [*r* = 0.840; Power = 0.99 (Figure 4H)].

The GLMM analyses revealed distinct effects of time, age, and covariates (FFM and DII) on cytokine levels. IL-1β showed no significant changes over time or due to the Time × Age group (Adolescent vs. Adult) interaction (Table 3). However, FFM emerged as a significant predictor, indicating that in both adolescents and adults, individuals with higher FFM presented with elevated levels of IL-1β.

**Table 3:** Generalized linear mixed models (GLMM) results for cytokine responses to repeated sprint exercise (RSE).

Cytokine	Omnibus fixed effects (*χ*^2^, df, p)	Fixed effect estimates [β (95% CI), SE, z, p]	Significant pairwise comparisons (Bonferroni-adjusted)	Simple effects (Time × Age group)
IL1β	- FFM (χ^2^ = 5.19, df = 1, *p* = 0.0023*); - Time (χ^2^ = 4.56, df = 3, *p* = 0.207); - Age group trend (χ^2^ = 3.25, df = 1, *p* = 0.072); - Time × Age group (χ^2^ = 3.43, df = 3, *p* = 0.330).	FFM: β=0.078 [0.011–0.145], SE = 0.034, *z* = 2.28, *p* = 0.023*.	There were no significant time contrasts.	There were no significant Time × Age group effects.
IL6	- Time (χ^2^ = 17.2, df = 3, *p* < 0.001*); - Time × Age group (χ^2^ = 8.3, df = 3, *p* = 0.040*); - FFM (*p* = 0.007*); - DII (*p* = 0.005*).	- Immediately after vs. 24h: *β* = 0.245 [0.087–0.403], SE = 0.081, *z* = 3.04, *p* = 0.002*; - Pre vs. 24h: *β* = 0.159 [0.033–0.285], SE = 0.064, *z* = 2.47, *p* = 0.014*.	- ↑ IL-6 immediately after and 2 h after vs. 24 h after (*p* < 0.05*); - Adults > Adolescents in Pre & 24 h after (*p* < 0.05*).	Adults showed stronger increases in IL-6 after and 24 h (*p* < 0.05*).
IL8	- Time (χ^2^ = 205.9, df = 3, *p* < 0.001*); - Age group (χ^2^ = 2.50, df = 1, *p* = 0.114); - Time × Age group (χ^2^ = 8.5, df = 3, *p* = 0.036*).	- 2 h vs. 24h: *β* = 0.072 [0.008–0.136], SE = 0.033, *z* = 2.19, *p* = 0.028*; - Immediately after vs. 24h: *β* = 0.331 [0.267–0.395], SE = 0.033, *z* = 10.14, *p* < 0.001*; - Pre vs. 24h: *β* = 0.389 [0.325–0.453], SE = 0.033, *z* = 11.93, *p* < 0.001*.	- All time points >24 h (*p* < 0.001*); - Adults > Adolescents (Pre & Immediately after).	Adults had higher IL-8 levels pre- and immediately after RSE (*p* < 0.05*).
IL10	- Time (χ^2^ = 1.139, df = 3, *p* = 0.767); - Age group (χ^2^ = 3.94, df = 1, *p* = 0.047*); - Time × Age group (χ^2^ = 23.493, df = 3, *p* < 0.001*).	- Time (2 h vs. 24 h): *β* = 0.0887 [0.04785–0.12954], SE = 0.0208, *z* = 4.2560, *p* < 0.001*; - Time (Immediately After vs. 24 h): *β* = 0.1088 [0.06792–0.14963], SE = 0.0208, *z* = 5.2181, *p* < 0.001*; - Age group (Adult - Adolescent): *β* = −0.1482 [−0.29459– −0.00188], SE = 0.0747, *z* = −1.9852, *p* = 0.047*.	- Adults: 2 h vs. 24 h (*p* < 0.001*); - Immediately After vs. 24 h (*p* < 0.001*); - Pre vs. Immediately After (*p* < 0.001*).	- Adults: significant time effects (χ^2^ = 20.08, df = 3, *p* < 0.001*); - Adolescents: no significant time effects (χ^2^ = 3.46, df = 3, *p* = 0.326).

IL, interleukyn; FFM, fat-free mass; ↑, increase; >, greater than; Group age, analysis of biological maturation when comparing adolescents [subjects in the process of maturation] with adults [biologically mature subjects].

For IL-6, there was a main effect of time and a significant Time × Age group interaction. *Post hoc* analyses showed that, in adults, IL-6 levels were significantly elevated immediately after exercise and 2 h later compared to the 24 h time point. Fixed effect estimates also indicated that adults had higher IL-6 levels than adolescents at the immediately after exercise and 24 h time points. FFM and DII were also significant predictors for this cytokine, with the effects applying to both groups.

IL-8 presented a strong time effect and a significant Time × Age group interaction. IL-8 levels were consistently higher at all pre and immediately after exercise time points compared to the 24 h time point, indicating a robust response to the effort. The interaction suggests that adults have consistently higher IL-8 levels before and after RSE compared to adolescents.

Finally, IL-10 did not show a main effect of time, but it did demonstrate a highly significant Time × Age group interaction. While adolescents showed no significant changes in IL-10 levels over time, adults exhibited a remarkable increase, with significantly higher levels 2 h and immediately after RSE compared to 24 h. This finding suggests that the anti-inflammatory response mediated by IL-10 is more effectively and acutely activated in adults in response to resistance exercise than in adolescents.

## Discussion

This study investigated the influence of FFM, BM, and the DII on cytokine responses to a repeated sprint exercise (RSE) protocol in male athletes. The findings revealed distinct associations between adolescent and adult groups, suggesting that the after exercise inflammatory profile appears to be modulated by FFM levels, BM stage, and dietary patterns.

### Inflammatory responses and fat-free mass

The results of this study demonstrate that FFM plays a crucial role in immunoregulation, as reflected in the modulation of cytokine levels in response to exercise (Table 3). Both group comparisons (Figures 2, 3 and Table 3) and correlation analyses (Table 1) indicate that individuals with higher FFM exhibit elevated IL-6 levels at rest and immediately after RSE. These findings may be attributed to the endocrine function of muscle tissue, which releases IL-6 in response to mechanical and metabolic stress (2). However, the negative correlation between FFM and IL-10 observed in adults (pre, 2 h, and 24 h after RSE) deserves special attention. Although IL-10 is classically considered an anti-inflammatory cytokine, its reduction in individuals with higher FFM may suggest a distinct immunological adaptation in adult athletes, in which there is a greater demand for acute inflammatory responses at the expense of chronic anti-inflammatory modulation. This hypothesis warrants further investigation (5, 44).

During adolescence, the growth and maturation of muscle tissue may influence inflammatory responses (14). This may serve as a metabolic adaptation and repair mechanism, which could explain why adolescents with higher FFM showed negative correlations with IL-6 and IL-8 at 24 h after RSE (Tables 1, 3), suggesting a more efficient inflammatory recovery (4). IL-8, a cytokine associated with neutrophil recruitment and maintenance of the inflammatory response (44, 50), may have been downregulated to prevent a prolonged inflammatory state in better-conditioned athletes (51, 52).

In contrast, adolescents with lower FFM showed a greater percent increase (Δ%) in IL-6 at 24 h after RSE compared to their peers (Figure 2B), suggesting that lower muscle reserves may compromise repair mechanisms and prolong the inflammatory response (2). Among adults, those with higher FFM consistently exhibited lower IL-10 levels (pre, 2 h, and 24 h after RSE) and a positive association between FFM and IL-6 at 2 h after RSE (Table 1), reinforcing the importance of additional regulatory mechanisms in the modulation of the inflammatory response (1, 4, 5). It is worth noting that the absence of significant correlations between FFM and IL-1β in adolescents may indicate that this cytokine is less sensitive to variations in body composition at this age or that other factors (e.g., training load or nutritional status) exert a greater influence (Tables 1, 3) (53).

### Inflammatory profile and biological maturation

When controlling for BM (Table 1), a positive association was observed between FFM and IL-10 levels, suggesting that adolescents with higher FFM may present an anti-inflammatory predisposition, regardless of maturation stage. This adjustment also revealed a similar correlation for IL-6 levels, indicating that individuals with more muscle mass exhibit an acute IL-6 response to exercise, reinforcing the role of muscle tissue as a potential source of this cytokine (Table 3) (1). In this context, the inverse correlation with IL-1β at 24 h after RSE in adolescents suggests that FFM may attenuate late inflammatory responses, possibly due to greater tissue repair capacity (44, 53).

Consistently, less mature adolescents showed a greater percent increase (Δ%) in IL-6 at 24 h after RSE compared to their more mature peers (Figure 3B), suggesting that inflammatory markers may be influenced by BM (10), possibly due to a still developing physiological system (14). Less mature individuals also tend to have lower FFM levels compared to their more biologically advanced peers (54), which may explain the similarity of findings when adolescents were grouped by FFM levels and BM status (Figures 2B, 3B). These findings may not be exclusively linked to lower FFM, but also to hormonal variations (e.g., testosterone and GH) that influence both muscle development and immune activity (53, 54).

Furthermore, adolescents with higher FFM appeared to exhibit lower IL-1β levels at 24 h after RSE (Table 1). IL-1β is a pro-inflammatory cytokine involved in initiating the inflammatory response and muscle soreness (44, 53). Lower concentrations at 24 h after RSE in individuals with higher FFM may indicate a more controlled inflammatory response and more efficient recovery, potentially associated with training-induced adaptations and greater anti-inflammatory capacity (1, 2).

### Inflammation and DII

The results of this study demonstrate that DII significantly influenced cytokine responses following RSE (Figures 4C,H). Specifically, lower DII scores (indicating a more anti-inflammatory dietary pattern) were associated with higher IL-8 levels in adolescents and higher IL-10 levels in adults. These findings align with literature showing an inverse relationship between anti-inflammatory diets and pro-inflammatory markers such as IL-1β, IL-6, IL-8, TNF-α, and C-reactive protein (CRP) (40, 41). The production of pro-inflammatory interleukins by muscle tissues during exercise is well documented and is considered an adaptive response that triggers anti-inflammatory pathways, mobilizes glycogen, and promotes metabolic adaptations (1, 55).

Among adults with higher FFM and anti-inflammatory dietary patterns, the observed increase in IL-10 after exercise may reflect enhanced anti-inflammatory responses. This aligns with previous evidence that IL-6 induces IL-10 and IL-1ra, modulating the inflammatory cascade (56, 57). Individuals with less inflammatory dietary patterns tend to present a more favorable redox and immune basal state, facilitating effective immune responses to physical stress (52, 58–61).

Individuals with less inflammatory dietary patterns tend to present a more favorable redox and immune basal state, facilitating effective immune responses to physical stress (14, 61). The DII reflects the inflammatory potential of various macronutrients and micronutrients, which directly affect cytokine levels (41). Diets rich in saturated fats, refined sugars, and low in fiber are associated with higher IL-6, IL-8, CRP, and TNF-α, whereas diets rich in fruits, vegetables, and omega-3 fatty acids promote anti-inflammatory responses, including IL-10 (40, 60).

Although the exact mechanisms remain to be clarified, it is presumed that an anti-inflammatory dietary pattern contributes to redox balance, reduced baseline expression of nuclear factor kappa B (NF-*κ*B), and lower secretion of pro-inflammatory adipokines (e.g., TNF-α, IL-1β), thereby enabling a rapid and transient IL-6 response to exercise without prolonged inflammation (62–64). During the recovery phase, the modulatory effect of DII is manifested through more efficient IL-10 expression, aiding inflammation resolution.

### Practical applications: insights for training load control

The findings of this study provide relevant insights for training load management, particularly in adolescent and adult athletes involved in intermittent sports. The observation that individuals with lower FFM exhibit greater inflammatory variation (especially IL-6 at 24 h after RSE) suggests a slower inflammatory recovery and a potentially higher risk of immune overload. This implies that athletes with lower muscle development may require longer recovery intervals between high-intensity sessions or complementary interventions (e.g., nutritional and sleep strategies) that support inflammation resolution.

In adolescents, the influence of BM on inflammatory responses reinforces the need to individualize training loads according to maturation status. Biologically less mature athletes showed more prolonged inflammatory responses (Figure 3B), which may hinder training adaptations. Monitoring PHV status, skeletal maturation, and puberty stages can help coaches and strength professionals avoid overloading immature athletes, promoting safer and more sustainable development.

In adults, greater FFM was associated with more effective IL-10 modulation, indicating a more favorable anti-inflammatory profile and enhanced recovery potential. This information may assist in adjusting training load based on athletes' morphological status, as individuals with higher FFM may tolerate greater volumes and intensities with lower risk of chronic inflammation.

Finally, the DII emerges as a practical and promising tool for sports science. Our findings indicate that dietary patterns with lower inflammatory potential (as reflected in lower DII values) are associated with more efficient immune responses to exercise, particularly in adults with higher FFM (greater IL-10) and adolescents (lower IL-8) (Figures 4C,H). This reinforces the need to incorporate regular nutritional assessments into training load monitoring. Nutritional intervention programs focused on healthy and anti-inflammatory diets, aimed at reducing DII, may serve as important complementary strategies. This nutritional approach seeks to reduce exercise-induced immune overload, optimizing recovery and sports performance across age groups and maturation stages.

### Limitations and future directions

Despite its relevant findings, the present study presents the following limitations: (i) Only athletes from intermittent sports were included, requiring caution when generalizing the findings to endurance sports; (ii) Only male athletes were analyzed, and extrapolation to female athletes should be made with caution; (iii) Although intragroup comparisons, statistical corrections, *post hoc* power analyses, and control for interaction variables (FFM, BM, and DII) were performed, caution is warranted when interpreting the results of the adult group due to the small sample size. We note that while the sample size and group imbalance are considerable limitations, the statistical methodology employed (GLMM) was chosen to minimize the impact of these factors, thereby increasing the internal validity of the results with the observation of 120 events when considering the time points (pre, immediately after, 2 h, and 24 h after RSE); (iv) Finally, a limitation to be considered is the absence of a control group for the RSE protocol; however, the repeated-measures design, combined with the GLMM analysis, allowed each participant to serve as their own control, strengthening the study's internal validity.

Future studies should seek to address the limitations outlined in this section.

## Conclusion

This study concludes that FFM, BM, and the DII significantly influence the inflammatory response to repeated sprint exercise (RSE) in male athletes. In adolescents, BM appears to modulate the interaction between FFM and interleukin dynamics after intense exercise. Lower FFM levels were associated with a more pronounced and prolonged IL-6 response, indicating a potentially greater risk of immune overload and the need for more cautious recovery strategies. In adults, greater FFM was linked to a more robust IL-10 response, suggesting a more efficient anti-inflammatory profile. DII, in turn, independently modulated the immune response, with less inflammatory dietary patterns associated with cytokines that favor recovery (e.g., IL-10).

These findings highlight the importance of an integrated approach to training load management that considers not only body composition and BM, but also athletes' dietary patterns. The incorporation of DII as a nutritional monitoring tool may represent a strategic advancement in the personalization of training and the promotion of safe and effective immunometabolic adaptations. It is recommended that strength and conditioning coaches, sports scientists, and nutritionists adopt a multidimensional approach that include regular assessments of FFM, BM, and DII in training and recovery planning. Integrating these markers into evaluation and planning processes may significantly enhance the personalization of sports interventions, contributing to athletes' health, performance, and long-term development.

## Data Availability

The datasets presented in this study can be found in online repositories. The names of the repository/repositories and accession number(s) can be found below: https://figshare.com/articles/dataset/Raw_Data_-_Cytokines_DII_FFM_BM_Sprints_/29506262.
